# Supplementary material for: Eating disorder treatment in routine clinical care: A descriptive study examining treatment characteristics and short-term treatment outcomes among patients with anorexia nervosa and bulimia nervosa in Germany and Switzerland
Source: PLoS One. 2023 Jun 30;18(6):e0280402. doi: 10.1371/journal.pone.0280402 (PMC10313003; doi:10.1371/journal.pone.0280402)
Supplement: Attachment — Submitted filename: response to reviewers.docx [file pone.0280402.s002.docx]

**Reviewers' comments:**

Reviewer's Responses to Questions

**Comments to the Author**

1. If the authors have adequately addressed your comments raised in a previous round of review and you feel that this manuscript is now acceptable for publication, you may indicate that here to bypass the “Comments to the Author” section, enter your conflict of interest statement in the “Confidential to Editor” section, and submit your "Accept" recommendation.

Reviewer #2: All comments have been addressed

Reviewer #3: All comments have been addressed

2. Is the manuscript technically sound, and do the data support the conclusions?

The manuscript must describe a technically sound piece of scientific research with data that supports the conclusions. Experiments must have been conducted rigorously, with appropriate controls, replication, and sample sizes. The conclusions must be drawn appropriately based on the data presented.

Reviewer #2: Yes

Reviewer #3: Yes

3. Has the statistical analysis been performed appropriately and rigorously?

Reviewer #2: Yes

Reviewer #3: Yes

4. Have the authors made all data underlying the findings in their manuscript fully available?

The [PLOS Data policy](http://www.plosone.org/static/policies.action#sharing) requires authors to make all data underlying the findings described in their manuscript fully available without restriction, with rare exception (please refer to the Data Availability Statement in the manuscript PDF file). The data should be provided as part of the manuscript or its supporting information, or deposited to a public repository. For example, in addition to summary statistics, the data points behind means, medians and variance measures should be available. If there are restrictions on publicly sharing data—e.g. participant privacy or use of data from a third party—those must be specified.

Reviewer #2: Yes

Reviewer #3: Yes

5. Is the manuscript presented in an intelligible fashion and written in standard English?

PLOS ONE does not copyedit accepted manuscripts, so the language in submitted articles must be clear, correct, and unambiguous. Any typographical or grammatical errors should be corrected at revision, so please note any specific errors here.

Reviewer #2: Yes

Reviewer #3: Yes

6. Review Comments to the Author

Please use the space provided to explain your answers to the questions above. You may also include additional comments for the author, including concerns about dual publication, research ethics, or publication ethics.

Reviewer #2: The authors addressed all my concerns very well.
I have one minor comment regarding the revision:I think it is confusing that Table 1 and Table 2 contain partly the same and partly different variables. The authors might also consider combining the information into 1 Table and report the 4 subgroups and the 2 total samples in 1 Table regarding all different variables from Table 1 and 2 (as in Table 5 and 6)

Response: As suggested, have combined Table 1 and 2 into one Table.

Reviewer #3: This revised paper is excellent, and all of my questions and concerns from previous Peer review has been addressed.

7. **Do you want your identity to be public for this peer review?**

Reviewer #2: No

Reviewer #3: **Yes:** Amanda Sainsbury Salis
